# Differences in periprosthetic bone mineral density tendencies with Types 1 and 3C stems in bipolar hemiarthroplasty following hip fracture

**DOI:** 10.1038/s41598-023-44311-3

**Published:** 2023-10-09

**Authors:** Jun-Ki Moon, Jae Youn Yoon, Jin Woong Jeon, Incheol Kook, Chul-Ho Kim

**Affiliations:** 1 Department of Orthopedic Surgery, Chung-Ang University Hospital, Chung-Ang University College of Medicine, Seoul, Republic of Korea; 2 Department of Orthopedic Surgery, Seoul Now Hospital, Anyang-si, Gyeonggido Republic of Korea; 3 Department of Orthopedic Surgery, Hanyang University Hospital, Seoul, Republic of Korea; 4 Department of Orthopedic Surgery, Asan Medical Center, University of Ulsan College of Medicine, 88 Olympic-ro 43-gil, Songpa-gu, Seoul, Republic of Korea

**Keywords:** Trauma, Translational research, Musculoskeletal system

## Abstract

The purpose of this study was to compare the periprosthetic bone mineral density (BMD) changes in the patients who underwent bipolar hemiarthroplasty (BPHA) for geriatric femoral neck fracture between two major different types of cementless femoral stems. A total of 93 patients (96 hips) who underwent BPHA for femoral neck fracture were categorized into two groups: Type 1 (42 patients, 44 hips), and Type 3C stem (51 patients, 52 hips). We investigated the annual follow-up trends of periprosthetic BMD at each Gruen zone during minimum postoperative 5-years; moreover, we compared the trends of periprosthetic BMD between both groups. The mean follow-up period was 7.1 years. In both groups, the overall BMD at the last follow-up had decreased compared with the baseline. In those with the Type 1 stem, BMD in the lateral femoral meta-diaphysis significantly decreased at 1-year follow-up after surgery. In those with Type 3C stem, BMD in the lateral femoral metaphysis postoperatively decreased after 3-years, whereas the BMD in the mediolateral femoral diaphysis drastically decreased postoperative 1-year period and plateaued thereafter. Different tendencies according to stem design were observed obviously in the postoperative BMD change of the proximal femur in patients who underwent BPHA for geriatric femoral neck fracture.

## Introduction

With population aging and the higher proportion of the older population, the incidence of geriatric hip fracture is increasing every year^1,2^. Bipolar hemiarthroplasty (BPHA) is a standard treatment for displaced femoral neck fracture in older patients^3^. The cementless stem has gained popularity in arthroplasty because of the advantages of a shorter operation time, decreased blood loss, and higher cost-effectiveness as compared with the cemented stem^4,5^. According to the fixation mode and implant geometry, cementless stems can be classified into 6 representative types^6^. The cementless stem was developed as a strategy to enhance the clinical longevity of implants in order to reduce the resorptive bone remodelling secondary to stress shielding^7^, which may induce aseptic loosening of the implant as a result of periprosthetic bone loss^8,9^. Stem design, surface coating, stem length, and the position of the stem can affect the peri-stem stress shielding^10^. However, predicting the post-arthroplasty pattern and extent of stress shielding individually remains unelucidated.

To evaluate the degree of remodelling of the periprosthetic bone, the periprosthetic bone density is measured with dual-energy X-ray absorptiometry (DXA), which is a highly reproducible technique for quantitative monitoring of the changes in bone density that occur after total hip arthroplasty (THA)^11^. Inaba et al.^12^ used DXA and reported significant differences in postoperative bone mineral density (BMD) loss in the proximal femur with the use of 3 types of uncemented stems. However, the post-BPHA periprosthetic BMD change in patients with geriatric hip fracture has never been reported. As geriatric hip fracture itself is strongly associated with osteoporosis, periprosthetic BMD change can exacerbate the clinical outcomes of older patients as compared with those of patients who undergo THA for the treatment of hip disease. Furthermore, differences in femoral stem design can affect the degree of post-BPHA periprosthetic BMD change. Therefore, it is important to document periprosthetic BMD changes for each type of femoral stem in patients with geriatric hip fracture.

In this study of patients who underwent BPHA for geriatric femoral neck fracture, we aimed to compare the periprosthetic BMD changes associated with 2 different types of cementless femoral stems: Type 1 and Type 3C. These are the most popular stem types used for treating femoral neck fractures. The type 1 stem is a single-wedge stem with an anteroposteriorly flat and mediolaterally wide design, along with a distally tapering wedge shape^13^. This stem type is designed to fit into the femoral canal between the medial and lateral cortical bone of the femoral metaphysis. Type 1 stems can provide good rotational stability due to mediolaterally wide shape in the proximal part. The surface from proximal 1/3 to 5/8 of the stem is generally coated. The type 3C stem is a rectangular, tapered stem with a rectangular cross-section. Stems of this type are mostly fixed at the femoral metaphyseal-diaphyseal junction to facilitate three-point fixations in the sagittal plane. Additionally, the rectangular cross-sectional design can provide good rotational stability by making it possible for its four corners to embed into endosteal bone. Unlike type 1 stems, type 3C stems have a surface treatment of grit-blasting their across entire length. Excellent clinical outcomes have been reported in association with both stem types, with stem survival rates of 96–99% over more than 20 years of follow-up^14,15^.

## Results

The results of the comparison of demographic variables between the 2 groups are shown in Table 1. There were no significant differences in sex, mean age, and body mass index (BMI) of patients (*p* = 0.397, *p* = 0.145, and *p* = 0.521, respectively). There was no significant difference in the baseline T-score of the hip BMD screened at admission (*p* = 0.129). A large proportion of patients received osteoporosis medication throughout the study period in the Type 3C stem group (*p* = 0.025), although the duration of osteoporosis medication used did not differ between the 2 groups (*p* = 0.286). Compared to the Type 1 stem group, a higher proportion of participants in the Type 3C stem group had Dorr Type C femurs (6.8% vs. 13.5%), although this did not constitute a significant intergroup difference in the Dorr type-based femoral geometry (*p* = 0.589).

**Table 1:** Patients demographics.

Variables	Type of femoral component		p value
	Type 1 stem	Type 3C stem	
Patients, n (hips)	42 (44 hips)	51 (52 hips)	
Male/female (%)	8/36 (18.2/81.8)	6/46 (11.5/88.5)	0.397
Age, yrs ± SD	82.2 ± 11.5	85.0 ± 7.3	0.145
BMI, kg/m^2^ ± SD	22.9 ± 3.1	22.4 ± 3.4	0.521
T-score of BMD at admission period ± SD	− 2.9 ± 0.7	− 3.1 ± 0.9	0.129
Osteoporosis medication (%)	33 (75)	48 (92.3)	0.025
IV bisphosphonate	22/33 (66.7)	35/48 (72.9)	0.623
Oral bisphosphonate	4/33 (12.1)	1/48 (2.1)	0.153
Denosumab	4/33 (12.1)	6/48 (12.5)	1.000
Teriparatide	0/33 (0)	1/48 (2.1)	1.000
Sequential therapy	3/33 (9.1)	5/48 (10.4)	1.000
Duration of osteoporosis medication, years ± SD	2.7 ± 1.4	2.6 ± 1.9	0.286
Dorr type (%)			0.589
Type A	12 (27.3)	14 (26.9)	
Type B	29 (65.9)	31 (59.6)	
Type C	3 (6.8)	7 (13.5)	

*BMD* bone mineral density, *BMI* body mass index, *IV* intravenous, *SD* standard deviation.

### Serial follow-up trends of periprosthetic BMD in each Gruen zone

#### Type 1 stem

**Figure 1:**
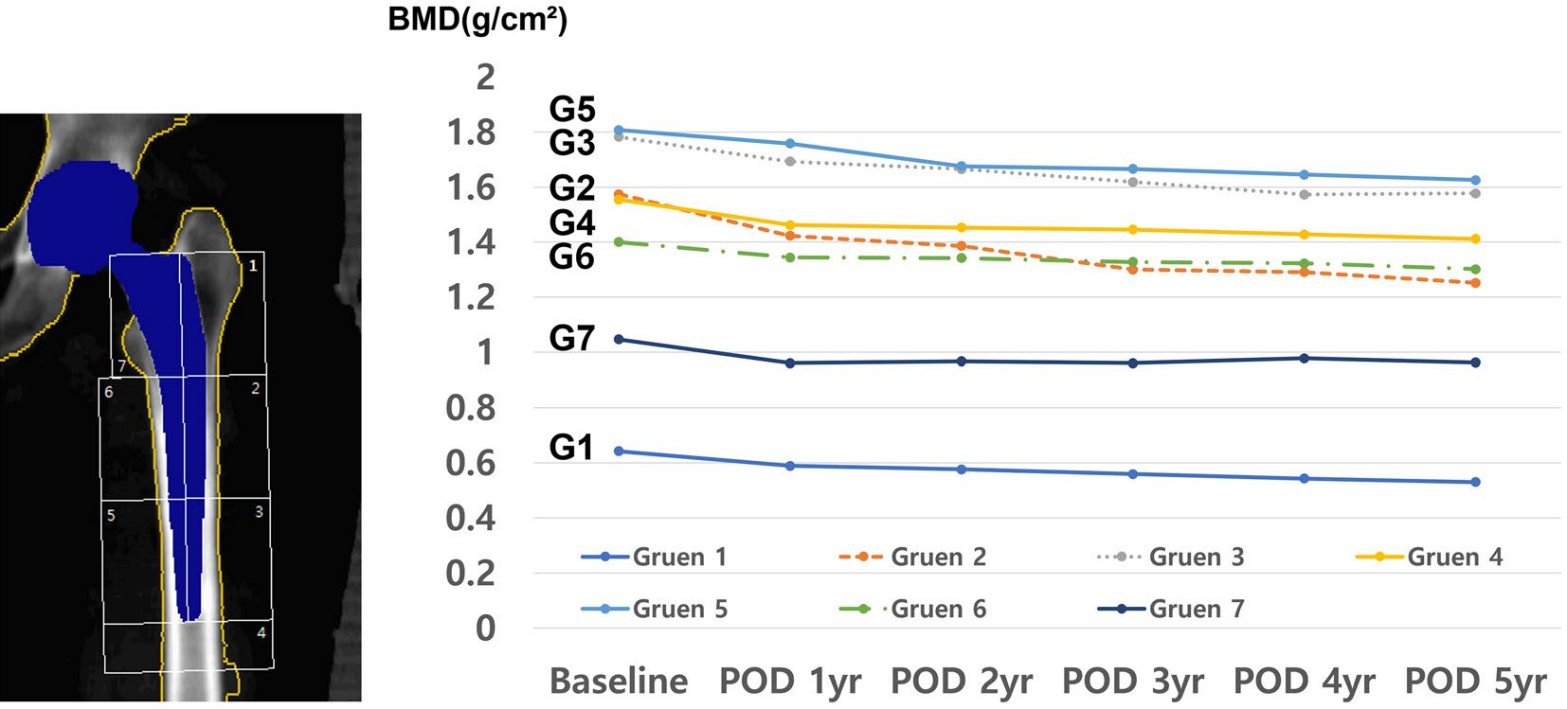
Serial trends of periprosthetic bone mineral density in the Gruen zones around the Type 1 stem.

The details of changes of BMD in Type 1 stem are described in Table 2 and line graphs were shown in Fig. 1. In Type 1 stem, the mean BMD significantly decreased in Gruen zones 1, 2, 3, and 4 at the postoperative 1-year period. In Gruen Zone 5, BMD was maintained for 1-year after surgery, and then significantly decreased at postoperative 2-year follow-up period. BMD in Gruen zones 6 and 7 slightly decreased during the overall follow-up period. The mean BMD values at the last follow-up in Gruen zones 1 to 5 were significantly lower than the baseline BMD value. Conversely, there was no significant difference in the BMD value in Gruen zones 6 and 7 between the baseline value and those at the last follow-up. Compared to the baseline, significant BMD reduction at the final follow-up was noted in Gruen Zone 1 (17.4% decrease) and Gruen Zone 2 (20.5% decrease).

**Table 2:** Serial follow-up trends of periprosthetic bone mineral density during 5-years after hip arthroplasty in Type 1 stem.

Area	f/u period	Mean BMD (g/cm^2^)	p value^†^	SD	Range
Gruen zone 1	Baseline	0.642		0.120	0.401–0.968
	POD 1 yr	0.589	0.033	0.145	0.273–0.834
	POD 2 yr	0.576	0.008	0.139	0.264–0.850
	POD 3 yr	0.559	0.001	0.121	0.252–0.703
	POD 4 yr	0.543	0.005	0.150	0.224–0.744
	POD 5 yr	0.530	0.003	0.130	0.226–0.656
*BMD change from baseline, Gruen Zone 1: 17.4% ↓					
Gruen zone 2	Baseline	1.574		0.198	1.104–1.957
	POD 1 yr	1.423	< 0.001	0.256	0.884–1.929
	POD 2 yr	1.386	< 0.001	0.282	0.671–1.834
	POD 3 yr	1.300	< 0.001	0.323	0.758–1.847
	POD 4 yr	1.291	0.001	0.245	0.796–1.546
	POD 5 yr	1.252	< 0.001	0.306	0.504–1.627
*BMD change from baseline, Gruen Zone 2: 20.5% ↓					
Gruen zone 3	Baseline	1.782		0.238	1.216–2.290
	POD 1 yr	1.693	0.007	0.286	1.039–2.304
	POD 2 yr	1.665	0.048	0.310	0.883–2.196
	POD 3 yr	1.618	0.001	0.316	1.049–2.050
	POD 4 yr	1.573	0.003	0.280	1.163–2.095
	POD 5 yr	1.577	0.008	0.270	1.107–1.904
*BMD change from baseline, Gruen Zone 3: 11.5% ↓					
Gruen zone 4	Baseline	1.554		0.232	1.010–2.025
	POD 1 yr	1.462	< 0.001	0.242	0.942–1.971
	POD 2 yr	1.453	0.034	0.261	0.852–1.816
	POD 3 yr	1.446	< 0.001	0.257	0.889–1.813
	POD 4 yr	1.428	0.001	0.258	0.983–1.840
	POD 5 yr	1.412	< 0.001	0.314	0.705–1.873
*BMD change from baseline, Gruen Zone 4: 9.1% ↓					
Gruen zone 5	Baseline	1.807		0.234	1.163–2.309
	POD 1 yr	1.758	0.304	0.274	0.987–2.362
	POD 2 yr	1.676	0.025	0.271	1.014–2.030
	POD 3 yr	1.666	0.009	0.300	0.954–2.088
	POD 4 yr	1.645	0.001	0.262	1.176–2.164
	POD 5 yr	1.625	0.007	0.292	1.197–2.111
*BMD change from baseline, Gruen Zone 5: 10.1% ↓					
Gruen zone 6	Baseline	1.400		0.239	0.832–1.954
	POD 1 yr	1.344	0.308	0.264	0.771–1.989
	POD 2 yr	1.342	0.291	0.262	0.855–1.955
	POD 3 yr	1.328	0.080	0.306	0.667–1.935
	POD 4 yr	1.323	0.068	0.236	0.980–1.780
	POD 5 yr	1.302	0.073	0.171	1.083–1.604
*BMD changes from baseline on Gruen zone 6: 7.0% ↓					
Gruen zone 7	Baseline	1.047		0.262	0.498–1.590
	POD 1 yr	0.961	0.109	0.293	0.578–1.710
	POD 2 yr	0.967	0.475	0.285	0.518–1.533
	POD 3 yr	0.961	0.529	0.299	0.413––1.400
	POD 4 yr	0.978	0.242	0.309	0.598–1.619
	POD 5 yr	0.963	0.143	0.216	0.628–1.280
*BMD change from baseline, Gruen Zone 7: 8.0% ↓					

*BMD* bone mineral density, *SD* standard deviation.

†Comparison with baseline values.

#### Type 3C stem

**Figure 2:**
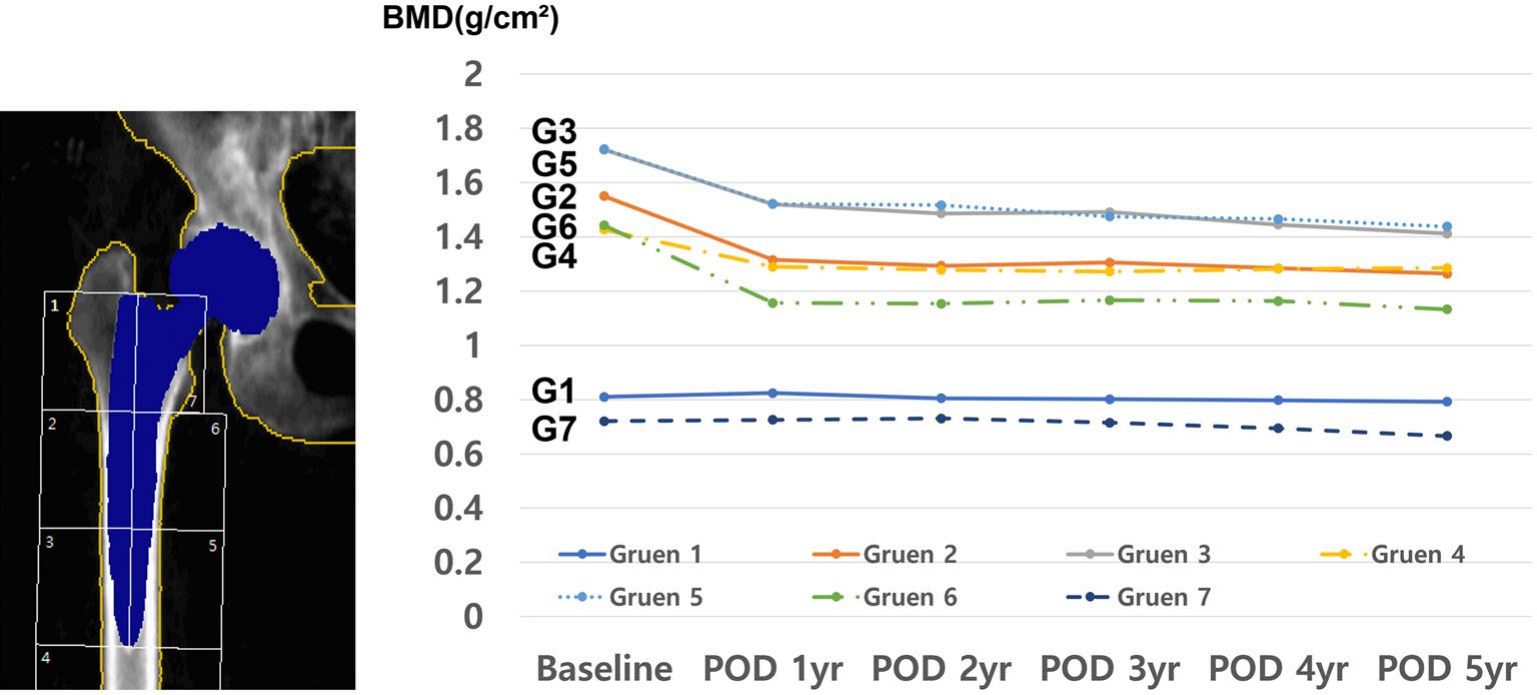
Serial trends of periprosthetic bone mineral density in the Gruen zones around the Type 3C stem.

The details of changes of BMD in Type 3C stem are described in Table 3 and line graphs are shown in Fig. 2. In Type 3C stem, the BMD changes showed 3 different trends. First, the BMD of Gruen Zone 1 was maintained for 3 years after surgery but significantly decreased thereafter. Second, the BMD of Gruen Zone 7 slightly decreased and was lower than those of other zones during the overall follow-up period. Third, the BMD value of Gruen zones 2–6 drastically decreased in the postoperative 1-year period, and showed a plateauing tendency after 2-years of the surgery. The mean BMD value at last follow-up in Gruen zones 1–6 was significantly lower than the baseline BMD value. There was no significant difference in the BMD value in Gruen Zone 7 from baseline to the last follow-up. Compared to the baseline, the most significant BMD reduction at the final follow-up was observed in Gruen Zone 6 (21.4% decrease).

**Table 3:** Serial follow-up trends of periprosthetic bone mineral density during 5-years after hip arthroplasty in Type 3C stem.

Area	f/u period	Mean BMD (g/cm^2^)	p value^†^	SD	Range
Gruen zone 1	Baseline	0.810		0.227	0.453–1.502
	POD 1 yr	0.824	0.483	0.203	0.494–1.282
	POD 2 yr	0.805	0.746	0.256	0.435–1.419
	POD 3 yr	0.801	0.084	0.304	0.332–1.426
	POD 4 yr	0.798	0.007	0.270	0.319–1.394
	POD 5 yr	0.792	0.001	0.239	0.301–1.208
*BMD change from baseline, Gruen Zone 1: 2.2% ↓					
Gruen zone 2	Baseline	1.550		0.263	1.067–2.163
	POD 1 yr	1.315	< 0.001	0.325	0.716–2.006
	POD 2 yr	1.293	< 0.001	0.320	0.733–1.848
	POD 3 yr	1.305	0.002	0.298	0.710–1.802
	POD 4 yr	1.284	< 0.001	0.318	0.619–1.632
	POD 5 yr	1.264	< 0.001	0.269	0.693–1.710
*BMD change from baseline, Gruen Zone 2: 18.5% ↓					
Gruen zone 3	Baseline	1.721		0.258	1.089–2.228
	POD 1 yr	1.520	< 0.001	0.297	0.875–2.316
	POD 2 yr	1.486	< 0.001	0.291	0.939–1.897
	POD 3 yr	1.491	0.001	0.250	0.944–1.825
	POD 4 yr	1.445	0.003	0.306	0.784–1.804
	POD 5 yr	1.412	0.001	0.316	0.712–1.776
*BMD change from baseline, Gruen Zone 3: 18.0% ↓					
Gruen zone 4	Baseline	1.427		0.262	0.967–1.939
	POD 1 yr	1.290	< 0.001	0.269	0.769–1.990
	POD 2 yr	1.278	< 0.001	0.290	0.768–1.801
	POD 3 yr	1.272	< 0.001	0.251	0.849–1.832
	POD 4 yr	1.282	0.001	0.324	0.693–1.729
	POD 5 yr	1.285	< 0.001	0.331	0.745–1.713
*BMD change from baseline, Gruen Zone 4: 10.0% ↓					
Gruen zone 5	Baseline	1.723		0.283	1.211–2.337
	POD 1 yr	1.522	< 0.001	0.301	0.757–2.190
	POD 2 yr	1.517	0.002	0.302	1.001–2.088
	POD 3 yr	1.475	0.005	0.229	0.851–1.810
	POD 4 yr	1.466	0.003	0.293	0.754–1.719
	POD 5 yr	1.438	< 0.001	0.332	0.714–1.798
*BMD change from baseline, Gruen Zone 5: 16.5% ↓					
Gruen zone 6	Baseline	1.442		0.308	0.520–2.084
	POD 1 yr	1.156	< 0.001	0.317	0.415–1.941
	POD 2 yr	1.153	< 0.001	0.313	0.563–1.619
	POD 3 yr	1.166	0.003	0.368	0.524–1.846
	POD 4 yr	1.163	0.004	0.352	0.466–1.648
	POD 5 yr	1.133	< 0.001	0.238	0.647–1.504
*BMD change from baseline, Gruen Zone 6: 21.4% ↓					
Gruen zone 7	Baseline	0.720		0.275	0.289–1.336
	POD 1 yr	0.725	0.846	0.276	0.228–1.270
	POD 2 yr	0.730	0.688	0.279	0.309–1.362
	POD 3 yr	0.715	0.661	0.225	0.332–1.233
	POD 4 yr	0.694	0.849	0.468	0.280–1.517
	POD 5 yr	0.666	0.072	0.248	0.290–1.009
* BMD change from baseline, Gruen Zone 7: 7.5% ↓					

*BMD* bone mineral density, *SD* standard deviation.

^†^Comparison with baseline values.

## Discussion

In patients who underwent BPHAs for geriatric femoral neck fracture, the present study compared the periprosthetic BMD changes between those who received the Type 1 single wedge stem and Type 3C tapered rectangular stem. In both groups, the BMD in the overall areas at the last follow-up period decreased as compared with the baseline BMD. The important finding of this study was that, according to stem design, different tendencies of postoperative BMD loss of the proximal femur were identified in patients who underwent BPHA for geriatric hip fracture. Theoretically, the BMD loss in a particular zone can indicate a deterioration of bone quality in that region^16^, which may be related to fracture risk. Therefore, when clinically evaluating patients according to the stem type that is used, closer attention to the regions where BMD loss is commonly observed is required. To the authors’ knowledge, the comparison of BMD change after BPHA for geriatric hip fracture between 2 major stem designs has never been reported as previous studies compared BMD change after THA according to stem designs^12^.

Geriatric hip fracture is a devastating event that has increasing importance worldwide because of population aging^17,18^. This injury is strongly associated with osteoporosis, which increases the risk of hip fracture from low-energy trauma. BPHA remains a reliable treatment option for geriatric hip fracture, especially for a displaced femoral neck fracture^19–21^. The femoral stems used in this study are the most popular types of stem that are used for the treatment of proximal femur fracture^22–24^.

Of note, when comparing the periprosthetic BMD changes of Type 1 stem and Type 3C stem, the most significant differences were observed in Gruen zones 1 and 6. In Gruen Zone 1, the Type 1 stem was associated with a 17.4% BMD reduction from the baseline, whereas the Type 3C stem was associated with only a 2.2% BMD reduction at the final follow-up. In contrast, in Gruen Zone 6, the Type 1 stem was associated with a BMD reduction of only 7.0% whereas the Type 3C stem was linked to a remarkable 21.4% BMD reduction. With different stem designs, the BMD change of each zone might vary according to the amounts of stress shielding and aging. The present study obviously showed a significant difference in the trends of BMD change between the two stems that were evaluated.

Single-wedge cementless stems are generally used for arthroplasty, and ensure reliable clinicoradiological long-term outcomes. Inaba et al.^12^ reported that BMD in the medial-proximal femur was maintained for 3 years after THA in the group that received the tapered wedge-type stem. In the present study, although BMD slightly decreased in overall follow-up period, the BMD in the medial proximal femur was maintained for 5 years after BPHA. This finding might be explained by the fact that geriatric hip fracture is characterized by aging-related progression of BMD loss. Therefore, BMD in the lateral femoral meta-diaphysis significantly decreased at 1 year after BPHA, which was consistent with the results of a previous study.

The clinical outcome with Type 3C stem was reportedly favourable with excellent implant survivorships^25^. However, the occurrence of cortical porosis around femoral component in about 30% of patient was reported in unstable intertrochanteric fractures using type 3C stems^22^. Furthermore, the present study showed that the BMD in Gruen zones 2–6 around the Type 3C stem drastically decreased in the postoperative 1-year period, and this decrease might be associated with the cortical porosis. Thereafter, the BMD change showed a plateauing tendency and was not progressive. Based on these findings, cortical porosis around the Type 3C stem seems to stabilize postoperatively after 2 years. Lee et al.^23^ reported that cortical porosis did not impair the stability of the femoral component in the short-term postoperative period, and our findings support these previous observations. Inaba et al.^12^ reported that the BMD in the lateral-proximal femur was maintained for 3 years after THA that used the Type 3C stems. This result of the present study is consistent with that of the previous report, as no significant difference in the BMD value in Gruen zone 7 from baseline to that at the last follow-up was noted.

Fokter et al.^26^ compared the BMD change according to the implant type and reported that BMD atrophy was shown in Gruen zone 1, 4 and 7 in bionic stem group. Bionic stems are shorter than conventional stems, and bionic stems have large lateral notches, which give the implants additional rotational stability. Better bone preservation in Gruen zone 7 could be expected, but there was a BMD decrease in Gruen zone 7 in the bionic stem group, probably due to the large lateral notch necessitating more extensive resection of the greater trochanter during stem implantation. In the present study, some implants used in the type 3C stem group had tapered lateral shoulders, which might require more bone broaching around the greater trochanter. However, based on the findings of the present study, this effect appears to have been minimal.

The present study had few limitations. First, this study has a retrospective design and a small sample. However, we strengthened the study design through strict inclusion and exclusion criteria and a minimum 5-year follow-up period of periprosthetic BMD values. This study included only BPHA patients. A considerable number of patients with fragility-induced hip fractures underwent THA, especially in the high-demand group. However, due to the surgeon’s preference, periprosthetic BMD measurements were not performed in these patients during the study period and, therefore, patients with fragility-induced hip fractures who underwent THA were excluded from this study. Owing to the limitation of the retrospective study design, we were unable to investigate recently introduced stem designs (e.g., ultra-short stem). Melisik et al.^27^ recently reported that an ultra-short cementless anatomical stem was associated with an appropriate bone trabecular development around the stem during a minimum 5-year follow-up period. Second, the present study did not investigate the clinical results, including patient-reported outcomes and complications. Further study will be required to reveal the associations between the BMD change around the stem and clinical outcomes or complications, such as a periprosthetic fracture. Third, osteoporosis medication use might be a confounder because the present study included both patients with and without osteoporosis. Furthermore, due to the retrospective nature of the study, we were unable to conduct a subgroup analysis based on the type of osteoporosis medication that was used. Fourth, within each type of group, the diversity in the manufacturers of the stems could potentially introduce bias. Particularly, for Type 3C stems, one type of implant was dominant, whereas Type 1 stems were procured from as many as five different manufacturing types, and the lack of an analysis by the stem origin constitutes a possible limitation.

In conclusion, different tendencies according to the stem design were observed in postoperative BMD changes in the proximal femur in patients who underwent BPHA for geriatric hip fracture. BMD in the lateral femoral meta-diaphysis (Gruen zones 1–4) significantly decreased at the 1-year post-BPHA follow-up with the Type 1 single-wedge stem. However, in those who received the Type 3C tapered rectangular stem, the BMD in the lateral femoral metaphysis (Gruen Zone 1) was maintained for 3 years following BPHA then significantly decreased. Based on these findings, type 3c stems may predominate in geriatric femoral neck fracture treatment, given the initial maintenance of BMD in the lateral femoral metaphysis. These differences should be considered when selecting cementless femoral stems according to the patient’s characteristics.

## Methods

This study was approved, and informed consent was waived due to the retrospective nature of the study and the analysis used anonymous clinical data, by the Ethics Committee of Institutional Review Board of Chung-Ang University Hospital, Seoul, Republic of Korea (IRB No. 2209-009-19435). All experiments were performed in accordance with relevant guidelines and regulations.

### Patient selection

We retrospectively reviewed the electronic medical records of all consecutive patients who underwent hip arthroplasty with DXA screening at admission in our institution from December 1, 2010 to June 30, 2017 due to geriatric hip fracture sustained with low-energy trauma (resulting from a fall from standing height or less) when aged ≥ 65 years. Patients who were diagnosed with a femoral-neck fracture, including basicervical fracture, were enrolled. We excluded patients who had fractures in the pertrochanteric region. We also excluded those who (1) underwent revision hip arthroplasties or (2) had a history of hip surgery on the ipsilateral hip. We included patients who (1) were followed up for at least 5 years postoperatively, with annual records of BMD-DXA measurement, and (2) underwent BPHA due to the fragility-related hip fracture, which resulted following a fall from standing height or less. The participants were classified according to the type of the femoral stem based on the classification that was developed by the Mont group^6^.

**Figure 3:**
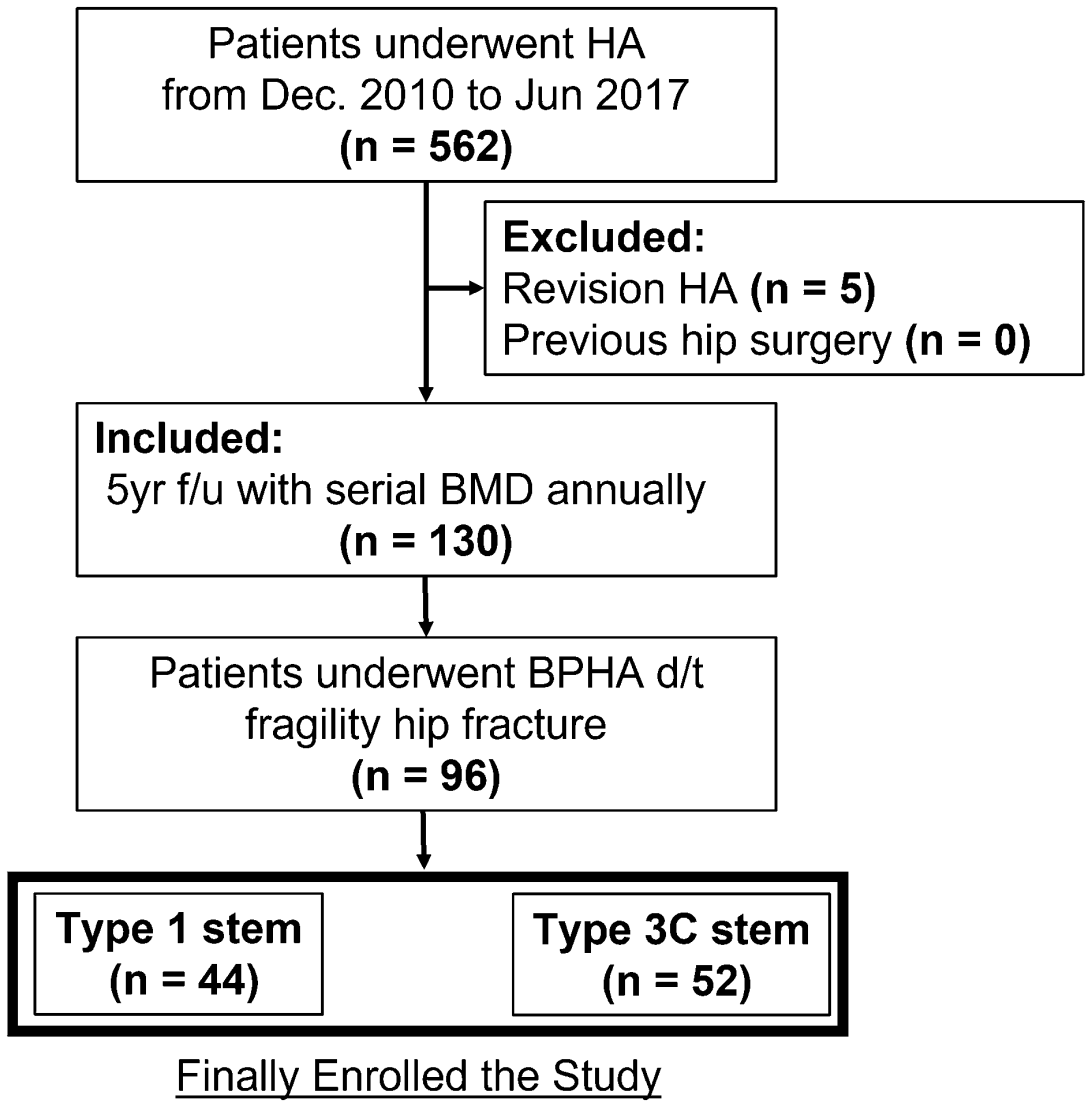
Flow diagram of the participant enrolment in the study.

Among the initially screened 532 patients (562 hips) who underwent HA and had baseline BMD-DXA examination data that were recorded during the study period, we excluded 5 patients who underwent revision hip arthroplasty. No patient had previously undergone hip surgery on the ipsilateral hip. Thus, we identified 124 patients (130 hips) who had serial follow-up data of BMD-DXA for at least 5-years after the surgery. Of these, we only included the 93 patients (96 hips) who underwent BPHA due to a geriatric hip fracture. Accordingly, 42 (44 hips) and 51 (52 hips) patients for whom a Type 1 or Type 3C stem, respectively, was used were finally enrolled (Fig. 3).

The mean follow-up period of study population was 7.1 (range 5.1–11.6) years. In the group with a Type 1 stem, 3/44, 1/44, 27/44, 10/44, and 3/44 patients received Taperloc^®^ (Zimmer-Biomet, Warsaw, IN, USA), Anthology^®^ (Smith & Nephew, Warsaw, IN, USA), Bencox M^®^ (Corentec, Seoul, Republic of Korea), Bencox ID^®^ (Corentec, Seoul, Republic of Korea), and Master SL^®^ (Lima Corporate, Udine, Italy) stem, respectively. In Type 3C stem, 35/52 were SL-PLUS MIA™ (Smith & Nephew, Warsaw, IN, USA), 1/52 was a C2 (Lima Corporate, Udine, Italy) stem, 1/52 was a Bencox II^®^ (Corentec, Seoul, Republic of Korea) stem, and the remaining 15/52 were Benfix^®^ (Corentec, Seoul, Republic of Korea) stem.

### Surgical details and perioperative management

All procedures were performed by one experienced faculty surgeon who specialized in adult hip reconstruction surgery. All surgeries were performed in the lateral decubitus position using the posterolateral approach.

During the study period, Taperloc^®^ (Zimmer-Biomet, Warsaw, IN, USA), Anthology^®^ (Smith & Nephew, Warsaw, IN, USA), Bencox M^®^ (Corentec, Seoul, Republic of Korea), Bencox ID^®^ (Corentec, Seoul, Republic of Korea), and Master SL^®^ (Lima Corporate, Udine, Italy) stems were used as Type 1 stems, whereas the SL-PLUS MIA™ (Smith & Nephew, Warsaw, IN, USA), C2 (Lima Corporate, Udine, Italy), Bencox II^®^ (Corentec, Seoul, Republic of Korea), and Benfix^®^ (Corentec, Seoul, Republic of Korea) stems were used as Type 3C stems. The postoperative rehabilitation program was the same for all patients; patients were allowed flat-foot partial weight-bearing with a walker or crutches on postoperative Day 2 and were allowed full weight-bearing as tolerated after 6-weeks of the surgery.

### Measurement of periprosthetic BMD in seven Gruen zones

During the study period, all of BMD measurement by DXA was performed using Lunar Prodigy Advance system (GE Healthcare, WI, USA). The periprosthetic BMD was measured in accordance with the following steps: (1) exclude the metal region from the scanned area by using the proprietary software that automatically highlights the bone area, after excluding the implant, in the images; thus, the bone area was identified and selected by the BMD program which was included with the BMD scanner; (2) the automatically selected area (the region of interests) was then manually revised by an experienced radiological technologist for all of the BMD data. The periprosthetic BMDs were measured in 7 areas around the stem that were divided as per the Gruen’s zone^28^ in anteroposterior view as follows: Zone 1 is the area that includes the greater trochanter, and has an upper margin that is delineated by a horizontal line from the centre of the implant head and a lower margin that is a horizontal line from the centre of the lesser trochanter; Zones 2 and 3 are divided equally in half between Zones 1 and 4. Zone 4 is the area below the tip of the stem. Zones 5 and 6 are divided equally in half between Zones 4 and 7. Zone 7 is the medial area above the lesser trochanter.

### Data collection

From the electronic medical records, we extracted the data of the follow-up period of the participants as well as the implant usage profile for the Type 1 and Type 3C stem groups.

To compare the demographics of the Type 1 and Type 3C stem groups, we collected data including sex, age, BMI, preoperative diagnosis for hip arthroplasty, type of surgery performed, and, as the baseline characteristic, the hip BMD T-score at admission, which was measured as the lowest value among the femoral neck, trochanter, and shaft, and total average value, excluding Ward’s triangle. Moreover, we ascertained the number of patients who were prescribed osteoporosis medication as well as the duration of osteoporosis medication use in the entire study period.

We investigated the serial follow-up trends of periprosthetic BMD at each Gruen zone, annually during the 5-year postoperative period in the Type 1 and Type 3C stem groups. Furthermore, we compared the trends of periprosthetic BMD in the 7 Gruen zones in the two stem-type-stratified groups.

The BMD was measured both at baseline (at admission) and postoperatively. For the demographic analysis stratified by hip BMD, we used measurements from the contralateral hip, which constitutes a representative value for determining the osteoporosis status. The baseline periprosthetic BMD was measured according to the Gruen zone classification on the operated side.

### Statistical analysis

To compare demographic variables between the two groups, the independent *t*-test was used for continuous variables, whereas the chi-square or Fisher’s exact test was used to evaluate categorical variables, after verifying the assumption that the data follows a normal distribution. As all continuous variables were normally distributed, the independent *t*-test were used to evaluate continuous variables in this study. To compare the periprosthetic BMD at each follow-up time point with the baseline value in each Gruen zone, paired *t*-tests were used. All statistical analyses were performed using PASW Statistics version 18.0 (IBM Corp., Armonk, NY, USA). A *p* < 0.05 was considered significant.

## Data Availability

The datasets used and/or analyzed during the current study are available from the corresponding author on reasonable request.
