# Author Correction: Differences in periprosthetic bone mineral density tendencies with Types 1 and 3C stems in bipolar hemiarthroplasty following hip fracture

**DOI:** 10.1038/s41598-024-65503-5

**Published:** 2024-06-26

**Authors:** Jun-Ki Moon, Jae Youn Yoon, Jin Woong Jeon, Incheol Kook, Chul-Ho Kim

**Affiliations:** 1 Department of Orthopedic Surgery, Chung-Ang University Hospital, Chung-Ang University College of Medicine, Seoul, Republic of Korea; 2 Department of Orthopedic Surgery, Seoul Now Hospital, Anyang-si, Gyeonggido Republic of Korea; 3 Department of Orthopedic Surgery, Hanyang University Hospital, Seoul, Republic of Korea; 4 Department of Orthopedic Surgery, Asan Medical Center, University of Ulsan College of Medicine, 88 Olympic-ro 43-gil, Songpa-gu, Seoul, Republic of Korea

Correction to: *Scientific Reports* 10.1038/s41598-023-44311-3, published online 09 October 2023

The Funding section in the original version of this Article was omitted. The Funding section now reads:

“This research was supported by the Chung-Ang University Research Grants in 2022.”

The original Article has been corrected.

